# Dietary sea cucumber cerebroside alleviates orotic acid-induced excess hepatic adipopexis in rats

**DOI:** 10.1186/1476-511X-11-48

**Published:** 2012-05-08

**Authors:** Bei Zhang, Changhu Xue, Xiaoqian Hu, Jie Xu, Zhaojie Li, Jingfeng Wang, Teruyoshi Yanagita, Yong Xue, Yuming Wang

**Affiliations:** 1 College of Food Science and Engineering, Ocean University of China, Qingdao, China; 2 Department of Applied Biological Sciences, Saga University, Saga, Japan

**Keywords:** Sea cucumber cerebroside, Orotic acid, Fatty liver, Lipogenesis, Microsomal triglyceride transfer protein

## Abstract

**Background:**

Nonalcoholic fatty liver disease (NAFLD) is a prevalent chronic liver disease in industrialized countries. The present study was undertaken to explore the preventive effect of dietary sea cucumber cerebroside (SCC) extracted from *Acaudina molpadioides* in fatty liver rats.

**Methods:**

Male Wistar rats were randomly divided into four groups including normal control group, NAFLD model group, and two SCC-treated groups with SCC at 0.006% and 0.03% respectively. The fatty liver model was established by administration of 1% orotic acid (OA) to the rats. After 10d, serum and hepatic lipid levels were detected. And the serum alanine aminotransferase (ALT) and aspartate aminotransferase (AST) activities were also determined. Besides, to gain the potential mechanism, the changes of key enzymes and gene expressions related to the hepatic lipid metabolism were measured.

**Results:**

Dietary SCC at the level of 0.006% and 0.03% ameliorated the hepatic lipid accumulation in fatty liver rats. SCC administration elevated the serum triglyceride (TG) level and the ALT, AST activities in OA-fed rats. The activities of hepatic lipogenic enzymes including fatty acid synthase (FAS), malic enzyme (ME) and glucose-6-phosphatedehydrogenase (G6PDH) were inhibited by SCC treatment. And the gene expressions of FAS, ME, G6PDH and sterol-regulatory element binding protein (SREBP-1c) were also reduced in rats fed SCC. However, dietary SCC didn't affect the activity and mRNA expression of carnitine palmitoyltransferase (CPT) in liver. Besides, suppression of microsomal triglyceride transfer protein (MTP) activity was observed in SCC-feeding rats.

**Conclusions:**

These results suggested that dietary SCC could attenuate hepatic steatosis due to its inhibition of hepatic lipogenic gene expression and enzyme activity and the enhancement of TG secretion from liver.

## Background

The metabolic syndrome, a cluster of metabolic abnormalities such as hyperlipidemia, diabetes mellitus, and hypertension, is a widespread disease in industrialized countries and contributes to the increase in cardiovascular morbidity and mortality [1-3]. Nonalcoholic fatty liver disease (NAFLD), an increasingly prevalent chronic liver disease in many countries, has been associated with insulin resistance and metabolic syndrome. NAFLD is the preferred term to describe the spectrum of liver damage ranging from hepatic steatosis to steatohepatitis, liver fibrosis, and cirrhosis [4]. It has been reported that occurrence of NAFLD ranges from 10% to 24% in different populations, and even reaches 74% in obese individuals [5]. This prevalence prompted studies to focus on the prevention or therapy of NAFLD. Though the processes through which steatohepatitis evolves from hepatic steatosis are not fully understood, it is necessary to develop effective therapies for the treatment of NAFLD and to discover nutrients that will reduce the risk of NAFLD.

Orotic acid (OA), an intermediate in pyrimidine biosynthesis, is known to evoke fatty liver when fed over an appreciable amount in rats [6,7]. The model established by OA-supplemented diet to rats reflects the natural etiologic setting in which NAFLD develops[8]. OA-induced NAFLD has been reported to be possibly generated, in part, by reduction of very low density lipoprotein (VLDL) secretion [9], enhancement of triglyceride (TG) synthesis [7,10] and decrease in fatty acid β- oxidation [8,11,12]. The hepatic TG accumulation caused by OA which has been demonstrated before was likely to be alleviated by dietary nutrients. Buang Y has found that dietay phosphatidylcholine alleviated OA-induced hepatic steatosis and hepatomegaly through downregulation of fatty acid synthesis and up-regulation of β-oxidation in rats [13]. Dietary saponins of sea cucumber attenuating fatty liver partly associated with the promoted lipolysis via PPARα activation and inhibited SREBP-1c-mediated lipogenisis [12]. But no study was undertaken to ascertain the influence of dietary glycolipids against NAFLD.

The existence of glycolipids has been determined in wide range. Early in 2000, Suzuki H has detected glycolipids from several plants [14]. Recent growing interest in the physiological functions of glycolipid as a nutrient in food has generated. The antifungal and antibacterial activities in the three sphingolipids isolated from cucumber *Cucumis sativus L*. has been evaluated recently [15].Glycolipids has been shown great anticancer potency through inducing Th1-biased cytokines and CD8/CD4 T cells in mice bearing breast and lung cancers [16]. Nakagawa R discovered that galactosylceramide activated NKT cells and then produced IFN-γ, which both increased the innate antitumor cytotoxicity of NK cells and expessed inhibition of tumor metastasis to liver through the adaptive antitumor response of CD8+ T cells in mice [17]. The effect of glycolipids on the immunomodulatory has also been investigated peviously[18]. The cerebroside, a type of endogenous glycolipid extensively existing in cell membranes of fungi, plants, animals and marine organism [19], has been proven to exert pharmacological effects, such as antibacterial, antihepatotoxic, anti-tumor and neuroprotective activities [20-23]. And the cerebroside was also reported to regulate lipid levels according to Cohn Jeffrey [24]. The marine cerebroside displayed strong bioactivity as a result of the special ocean circulation, although there are fewer classes and lower contents of cerebrosides in marine than living in the ground. Sea cucumber of Echinodermata is an important marine food and medical material in Asian countries. Researches on the influence of dietary whole sea cucumber on lipid metabolism have been much investigated. Tanaka K has found that dietary 5% black sea cucumber (*Stichophus japonicus*) revealed hypolipidemic effect in rats [25]. The results of Wang's study on sea cucumber (*Pearsonothuria graeffei* and *Apostichopus japonicus*) displayed its lipid-lowering effect in hypercholesteremic rats [26]. However, it is unknown whether the sea cucumber cerebroside (SCC) may prevent against lipid metabolism disorder in animal models.

Whereas the SCC may be expected to exhibit various physiological functions based on the results obtained from cerebrosides from other sources. Nonetheless, reports on biological evaluation of lipid metabolism on SCC are unavailable. Therefore, the present work was carried out to discuss the effect of SCC on the lipids of serum and liver in rats fed OA diet. To gain an insight into the mechanism of SCC on the NAFLD caused by OA, response of enzymatic activities and mRNA expression involved in hepatic lipid metabolism was also measured.

## Methods

### Isolation and purification of cerebroside

**Figure 1:**
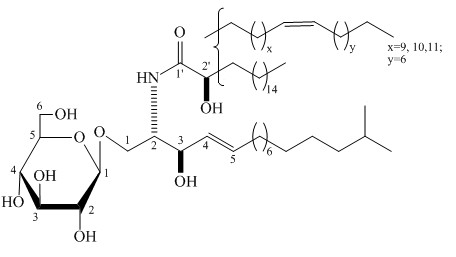
**The structure of SCC from *Acaudina molpadioides.***

The dried body wall of sea cucumber *Acaudina molpadioides* was grinded to powder and extracted three times with EtOH/H_2_O (4:1). The combined extracts were concentrated in vacuo to give an aqueous solution. The aqueous solution was extracted with three portions of n-hexane. The organic layer was concentrated in vacuo and the residue was washed with cold acetone to give an acetone-insoluble fraction. The acetone-insoluble part was chromatographed on Silica gel column(solvent CHCl_3_/MeOH 99:1 to CHCl_3_/MeOH/H_2_O 80:15:1) to afford cerebrosides (purity ≥ 95%) [27]. The structure of the SCC is shown in Figure 1.

### Animals and diets

All aspects of the experiment were conducted according to guidelines provided by the ethical committee of experimental animal care at Ocean University of China (OUC, China).

Male Sprague–Dawley rats aged 5wk were purchased from Vital River (Beijing, China) and housed individually in an air-conditioned room with a 12-h light/dark cycle, a constant temperature of 24°C and relative humidity of 65 ± 15%. After a 1-wk adaptation period, rats were randomly divided into four groups (n = 6). The animals were then assigned to the following four different diets: 1) AIN-93 G diet (Cont); 2) AIN-93 G diet with 1% OA contained (OA); 3) 1% OA-contained AIN-93 G diet plus 0.006%SCC (OA + 0.006%); 4) 1% OA-contained AIN-93 G diet plus 0.03% SCC (OA + 0.03%). Basal experimental diets were prepared according to the recommendations of the American Institute of Nutrition (AIN). The composition of the diets and SCC concentration were shown in Table 1. Rats were allowed free access to water and food for 10 days. At the end of the feeding period, all rats were killed by bleeding from the abdominal aorta under diethyl ether anesthesia after overnight fasting. Serum was separated from the whole blood by centrifugation at 1500*g for 10 min at 4°C. Liver, perirenal adipose and epididymal adipose were immediately excised for analysis.

**Table 1:** Composition of experimental diets (g/kg diet)

**Ingredients**	**Cont**	**OA**	**OA + 0.006 **	**OA + 0.03 %**
Casein	200	200	200	200
Cornstarch	500	490	489.94	489.7
Sucrose	100	100	100	100
Corn oil	100	100	100	100
Mineral mix (AIN-93 G)	35	35	35	35
Vitamin mix(AIN-93 G)	10	10	10	10
Choline bitartrate	2	2	2	2
Cellulose	50	50	50	50
L-cystine	3	3	3	3
OA	—	10	10	10
SCC	—	—	0.06	0.3

Vitamin mixture (AIN-93VX; DyetsInc.).

Mineral mixture (AIN-93 G; DyetsInc.).

### Serum biochemistry analysis

Serum total cholesterol (TC), TG and low density lipoprotein cholesterol (LDL-c) were measured using enzymatic reagent kits from Biosino (Beijing, China) according to the manufacturer's instructions. Serum ALT and AST activities were determined using enzymatic kits from Njjctech (Nanjing, China).

### Measurement of hepatic lipid levels

Hepatic lipids were extracted and purified according to the method of Folch [28] and then dissolved with 10% Triton X-100. The concentrations of TG and TC in liver were detected using enzymatic reagent kits from Biosino(Beijing, China), and the hepatic phospholipid (PL) levels were determined according to the methods of Bartlett [29].

### Preparation of liver subcellular fractions and assays of hepatic enzyme activities

Portions of the fresh livers from individual rats were homogenized in ice-cold 0.25 M sucrose solution containing 10 mM Tris–HCl buffer (pH7.4) and 1 mM EDTA. After precipitating the nuclei fraction, the supernatant was centrifuged at 10,000*g for 10 min at 4°C to obtain mitochondria, which was used for carnitinepalmitoyl transferase (CPT) activity as described previously [30]. The resulting supernatant was re-centrifuged at 125,000*g for 60 min,the supernatant (cytosol) was used for the assays of fatty acid synthase (FAS), malic enzyme (ME) and glucose-6-phosphatedehydrogenase (G6PDH) activities as described [30-32], and the precipitate(microsomes) was used for the assays of microsomal triglyceride transfer protein (MTP) activity with an MTP assay kit from Roar Biomedical (New York, US). The protein concentration was determined according to the method of Lowry [33], with bovine serum albumin used as the standard.

### Determination of mRNA expression of hepatic genes

For analysis of gene expression, total cellular RNA was extracted from 100 mg frozen liver samples using the Trizol reagent (Invitrogen, USA) according to the manufacturer’s recommended procedures. RNA concentrations were determined spectrophotometrically and 1 μg total RNA was reverse transcribed using an MMLV reverse transcriptase kit (Promega, USA) and random primers (TOYOBO, Japan) in a reaction volume of 50uL for cDNA synthesis. The concentration of cDNA was analyzed by real-time detection PCR (ABI Prism 7500 Sequence Detection System, USA) using Sybr Green I Master Mix (TOYOBO, Japan). PCR was carried out with a final volume of 30 μL reaction mixture containing:15 μL master mix, 2 μL first-strand cDNA, 1 μL primer with each forward and reverse, and H_2_O to make up 30 μL. A dilution curve from one cDNA source using dilutions of 1:2, 1:4, 1:8 and a no-template control was run for each gene including sterol- regulatory element binding protein (SREBP-1c). The gene expression was determined by relative quantification using the standard curve method. A final melting curve guaranteed the authenticity of the target product. The expression signal of the house keeping gene ß -actin served as an internal control for normalization.The primer sequences used for real-time PCR were shown in Table 2.

**Table 2:** Primers used in this study

**Gene**	**Forward/reverse primer**	**Accession No.**	**Product length**
β-actin	GCAGATGTGGATCAGCAAGC GTCAAAGAAAGGGTGTAAAACG	NM_031144	111 bp
FAS	GGAACTGAACGGCATTACTCG GCCCAAACCCCATTTTCTA	X62888	153 bp
ME	TCACCTGCCCTAATGTCCCT CATGCCGTTATCAACTTGTCC	NM_012600	185 bp
G6PDH	GTTTGGCAGCGGCAACTAA GGCATCACCCTGGTACAACTC	NM_031559	108 bp
SREBP-1c	CGCTACCGTTCCTCTATCAA TTCGCAGGGTCAGGTTCTC	AF286470	166 bp
CPT1a	GCTTCCCCTTACTGGTTCC AACTGGCAGGCAATGAGACT	NM_012930	115 bp

Gene-specific primers were designed by Primer Premier 5.0 software.

### Statistical analyses

All the values are expressed as mean ± standard error of the mean of six rats. All statistical analyses were performed using PC SAS software. One-way ANOVA and Tukey’s post hoc test with least significant difference were used to compare group means. Homogeneity of variances was tested by Levene’s test and Welch’s ANOVA was used to compare group means when the group variances were unequal. P < 0.05 was considered statistically significant.

## Results

### Body weight gain, food intake, liver weight and adipose weight

As described in Table 3, the initial body weight (BW) was similar among the four groups. The amount of food consumed and food intake were almost the same in the period of experiment. OA feeding markedly elevated the liver weight compared to the controls (P < 0.01), while the rats fed with SCC resulted in a significant decrease in liver weight as compared with the OA-feeding rats (P < 0.05). There was no statistical difference among the four groups in the adipose tissue weight although dietary SCC slightly reduced the weight of perirenal adipose.

**Table 3:** Effect of SCC on body weight gain, food intake, liver and adipose weights in rats

	**Cont**	**OA**	**OA + 0.006 %**	**OA + 0.03 %**
Initial BW, g	160 ± 7	160 ± 6	159 ± 8	158 ± 7
Final BW, g	248 ± 9	242 ± 8	238 ± 5	236 ± 9
Food intake, g/d	13.9 ± 0.3	13.8 ± 0.3	13.5 ± 0.7	13.2 ± 1.6
Liver index, g/100 g BW	3.77 ± 0.3	4.65 ± 0.4^**^	4.15 ± 0.3^#^	4.05 ± 0.2^#^
Perirenal adipose, g/100 g BW	0.85 ± 0.11	0.89 ± 0.15	0.84 ± 0.17	0.76 ± 0.21
Epididymal adipose,g/100 g BW	0.87 ± 0.04	0.90 ± 0.06	0.84 ± 0.12	0.87 ± 0.23

Rats were fed the experimental diets for 10d.

Values are expressed as mean ± standard error of the mean of six rats. ^**^P < 0.01 , different from the control group; ^#^P < 0.05, different from the OA group.

### Serum and hepatic parameters

Table 4 summarized the lipid levels of the rats in serum and liver after 10 days of experiment. As compared with the controls, the serum TG and TC concentrations decreased (P < 0.05, P < 0.05) in the rats fed with OA, while the LDL-c level tended to decrease without significant difference. The rats in both SCC groups had higher serum TG content than the OA-fed rats (P < 0.05, P < 0.05) , but the TC and the LDL-c concentrations, although tended to reduce, were of no statistic difference in the two groups contrasting the OA group.

**Table 4:** Effect of SCC on serum and hepatic parameters in rats

	**Cont**	**OA**	**OA + 0.006 %**	**OA + 0.03 %**
Serum lipids, mmol/L				
TG	1.44 ± 0.14	0.69 ± 0.12^*^	1.25 ± 0.09^#^	1.20 ± 0.13^#^
TC	1.75 ± 0.13	1.47 ± 0.16^*^	1.54 ± 0.12	1.52 ± 0.10
LDL-c	1.53 ± 0.11	1.07 ± 0.15	1.19 ± 0.12	1.21 ± 0.13
Hepatic lipids, umol/g				
TG	15.8 ± 1.3	97.3 ± 3.5^**^	31.2 ± 6.7^#^	15.7 ± 2.9^##^
TC	19.2 ± 0.5	26.7 ± 3.2^*^	17.5 ± 2.1^#^	14.3 ± 0.7^##^
PL	34.5 ± 1.2	32.7 ± 1.3	33.2 ± 1.3	32.7 ± 1.5
Enzyme activities, IU/L				
ALT	9.21 ± 2.01	28.7 ± 5.13^**^	15.7 ± 1.76^#^	15.9 ± 1.93^#^
AST	22.0 ± 1.76	39.5 ± 3.65^**^	29.4 ± 1.01^#^	28.1 ± 1.65^#^

Rats were fed the experimental diets for 10d.

Values are expressed as mean ± standard error of the mean of six rats.

^*^P < 0.05, ^**^P < 0.01, different from the control group; ^#^P < 0.05,^##^P < 0.01, different from the OA group.

OA-feeding rats had a hepatic TG level four times higher than that in the controls (P < 0.01). But SCC supplementation at 0.006% and 0.03% in rats both attenuated the OA-induced TG accumulation in contrast to the OA feeding (P < 0.05, P < 0.01). The same tendency was observed in the hepatic TC level between the control and OA groups (P < 0.05); dietary SCC at both doses reduced the TC concentration (P < 0.05, P < 0.01) in comparison with the OA diet. The PL content was of no marked difference among the four groups.

Besides, rats fed OA diet displayed severe NAFLD-accompanied liver injury. The activities of ALT increased about 2-fold in the OA group as compared with the controls (P < 0.01) and the AST increased over 70% (P < 0.01). Administration of SCC at both doses ameliorated the hepatic damage. Dietary SCC at 0.006% reduced the ALT and AST activities by 45.3% and 25.6% respectively (P < 0.05, P < 0.05) and SCC at 0.03% by 44.6% and 27.6% separately (P < 0.05, P < 0.05).

### Hepatic enzyme activities involved in lipogenesis metabolism

**Figure 2:**
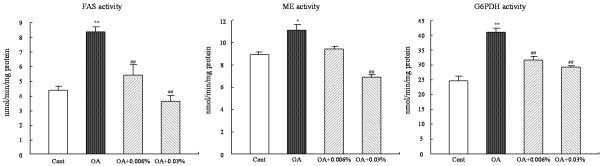
**Effect of SCC on the activities of the enzymes related to fatty acid biosynthesis in rats. **The activities of hepatic enzyme FAS, ME and G6PDH in rats fed Cont or OA-supplemented diet or the diet with OA + 0.006% SCC or OA + 0.03% SCC for 10d. Values are expressed as mean ± standard error of the mean of six rats. ^*^P < 0.05, ^**^P < 0.01, different from the control group; ^##^P < 0.01, different from the OA group.

OA administration increased the activities of FAS (89.8%, P < 0.01), ME (24.6%, P < 0.05) and G6PDH (72.8%, P < 0.05) in liver, while feeding SCC to NAFLD rats significantly abolished the OA-induced increase. Dietary SCC at 0.006% reduced the activity of FAS by 36.5% (P < 0.01) and G6PDH by 26.0% (P < 0.01), and tended to decreased the activity of ME. Addition of SCC at 0.03% significantly reduced the activities of FAS, ME and G6PDH by 59.1% (P < 0.01), 42.8% (P < 0.01) and 30.9% (P < 0.01) (Figure 2).

### Relative mRNA expression of SREBP-1c and its target genes in liver

**Figure 3:**
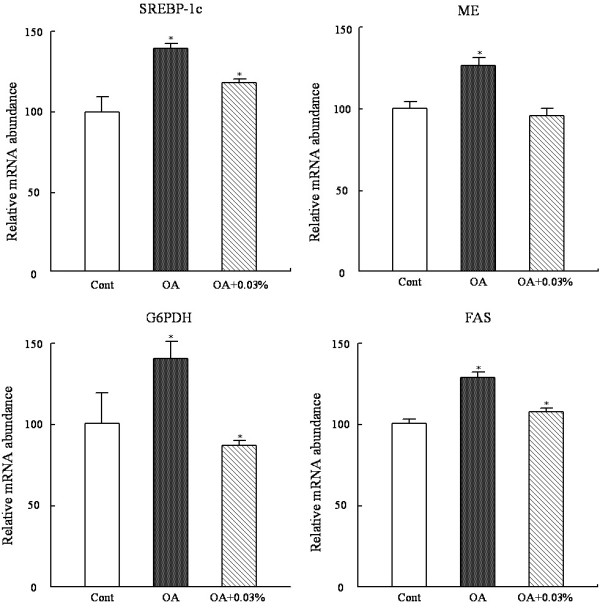
**Effect of SCC on the mRNA expression of lipogenic genes in the liver of the rats. **The mRNA expression of genes involved in lipogenesis SREBP-1c, FAS, ME and G6PDH in rats fed Cont or OA-supplemented diet or the diet with OA + 0.03% SCC for 10d. Values are expressed as mean ± standard error of the mean of six rats. ^*^P < 0.05 , different from the control group; ^#^P < 0.05, different from the OA group.

In order to explore the molecular mechanism of the effect of SCC on the regulation of fatty acid lipogenesis, the expression of hepatic lipogenic genes was examined (Figure 3). Considering the integrated lipid-lowering effects of SCC, the group by dietary 0.03% SCC was chosen to access the changes of the mRNA expression for its most effective actions.

**Figure 4:**
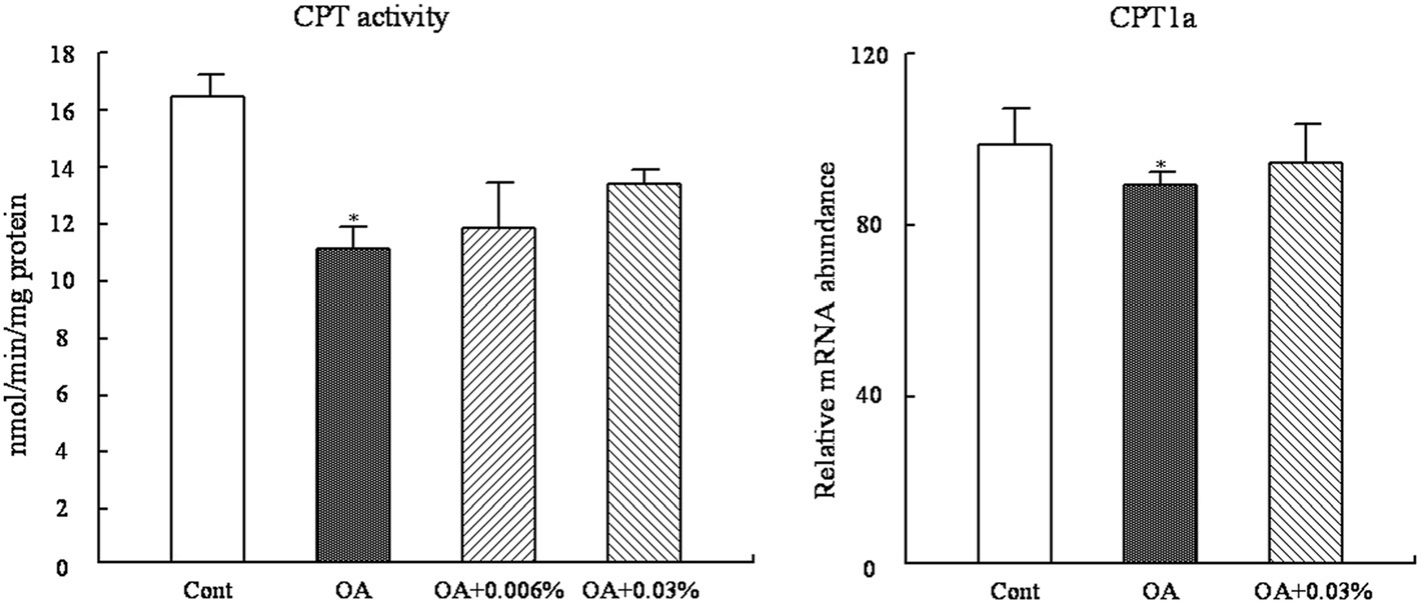
**Effect of SCC on the CPT enzyme activity and mRNA expression in the liver of the rats. **The activtiy of CPT in rats fed Cont or OA-supplemented diet or the diet with OA + 0.006% SCC or OA + 0.03% SCC and the mRNA expression of gene CPT1a in rats fed Cont or OA-supplemented diet or the diet withOA + 0.03% SCC for 10d. Values are expressed as mean ± standard error of the mean of six rats. ^*^P < 0.05 , different from the control group.

OA-supplemented diet to the rats up-regulated the mRNA expression of the lipogenic transcription factor SREBP-1c and its target genes (Figure 4). As compared with the controls, OA group exhibited a 1.4-fold SREBP-1c mRNA expreesion (P < 0.05), 1.3-fold FAS (P < 0.05) and 1.4-fold G6PDH (P < 0.05); and the expression of ME mRNA tended to increase without significant difference. Dietary SCC at 0.03% prevented the OA-stimulated up-regulation of SREBP-1c and its response genes. SCC supplementation down-regulated the mRNA expression of FAS, G6PDH and ME by 17.2% (P < 0.05), 43.0% (P < 0.05) and 24%(P < 0.05).

### Hepatic CPT activity and mRNA expression

As depicted in Figure 4, 1% OA addition reduced the activity of CPT, a key enzyme in fatty acid β-oxidation, by 37.1%(P < 0.05) as compared with the basal diet; The SCC at both doses tended to attenuate the reduction with no statistical difference. In line with the previous results, SCC at 0.03% was selected for considering the relative mRNA concentration of CPT1a. And consistent with the change of CPT activity, the CPT1a mRNA expression reduced 11% (P < 0.05) due to the supplemented-OA, but the SCC at 0.03% administration didn't affect its expression over the OA diet.

### Hepatic MTP activity

**Figure 5:**
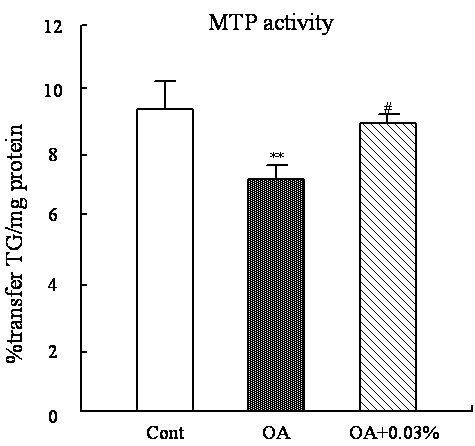
**Effect of SCC on the hepatic MTP activity in rats. **The activity of hepatic MTP in rats fed Cont or OA-supplemented diet or the diet with OA + 0.03% SCC for 10d. Values are expressed as mean ± standard error of the mean of six rats. ^**^P < 0.01 , different from the control group; ^#^P < 0.05, different from the OA group.

To gain further insight into the relationship of ipid metabolism between the liver and the serum, the activity of MTP was detected (Figure 5). OA feeding markedly inhibited the MTP activity (26.8%, P < 0.01), and SCC at 0.03% significantly attenuated the inhibition by 30.2% (P < 0.05) in contrast to the OA diet.

## Discussion

OA administration is widely used in studies of the mechanism of NAFLD. It is generally accepted that the enhancement of de novo lipid synthesis and access of hepatic fatty acids and inhibition of VLDL assembly and secretion result in OA-induced hepatic lipid accumulation [34]. The main objective of this study was to clarify if SCC contributed to NAFLD and to explore the potential mechanism in order to discover whether there is a new nutrient that prevents the development of NAFLD.

In the present study, dietary OA increased hepatic TG content by 4-fold, indicating the establishment of NAFLD model (Table 4). However, the raise in the hepatic TG concentration in rat was markedly reduced by simultaneous feeding of SCC at the dose of 0.006% and 0.03%. The SCC at 0.03% decreased the lipid level to a much lower extent. The same tendency was observed in hepatic TC levels. The results above suggested that dietary SCC improved hepatic lipid accumulation in a dose–response manner.

In addition, OA resulted in hepatic lipid accumulation and the increase in the activity of serum ALT and AST (Table 4). This indicated OA-induced NAFLD accompanied hepatic injury in rats. Nonetheless dietary SCC obviously attenuated the NAFLD-caused liver damage in rats via the declination of ALT and AST activities. Those results were in agreement with Zigmond E, who has found thatβ-glycosphingolipids alleviated liver damage through the immunomodulatory qualities inhibited local inflammation in the response organ in Cohen diabetic rats [35]. And to ourknowledg, this is the first report on the beneficial effect of SCC on NAFLD and concomitant liver injury in rats.

Several metabolic factors were involved in the hepatic lipid accumulation and the stimulation of hepatic fatty acid synthesis was the critical pathway of the NAFLD development. Therefore, the activities of the enzyme related to fatty acid biosynthesis were detected. And from Figure 1, a marked increase in the activity of FAS, a key enzyme involved in fatty acid synthesis, by OA diet was observed, and the activities of NADPH-generating enzymes required for FAS, ME and G6PDH, were also increasingly altered by OA-feeding, which was accordant with the study of Hu [12]. The present results demonstrated that SCC intake significantly suppressed the activities of those lipogenic enzymes, so one of the possible lipid-lowering mechanisms of SCC may be the inhibition of fatty acid biosynthesis.

As suggested in many researches, SREBP-1c acts a major part in the pathogenesis of NAFLD. The genes containing sterol-regulatory elements in their promoter regions in the lipogenic pathway are regulated by SREBP-1c including FAS, G6PDH and ME [36,37]. Over expression of SREBP-1c produces a pronounced hepatic TG accumulation leading to the development of NAFLD [38,39]. Thus, the depression of SREBP-1c expression in liver may potentially be responsible for the reduction of hepatic TG accumulation. As Figure 2 presented, SREBP-1c mRNA expression was strongly stimulated by OA diet, and supplementation of SCC inhibited its expression. And accordingly, the mRNA expressions of FAS, ME and G6PDH were concomitant markedly decreased in rats fed by simultaneously OA and SCC. Hence, the pathway of SCC-inhibition of lipogenis was supposed that SCC supplementation directly affected the SREBP-1c expression and then the alternation of these lipogenic gene transcriptions occur, thus reducing the activities of the responding enzymes. Another factor in charge of OA-induced hepatic TG accumulation is a declination of fatty acidβ-oxidation [12,40]. Since the total β-oxidation critically depends on the mitochondrial oxidation, the activity of the rate-limiting enzyme of mitochondrial β-oxidation, CPT, was examined to reflect the β-oxidation ability (Figure 4). Consistent with Miyazawa S [41], dietary OA significantly decreased the hepaticβ-oxidation capacity. However, SCC intake showed no significantly effect on the key enzyme activities, although a slight increase was observed by dietary SCC at both doses, especially at 0.03%. The result of CPT1a mRNA expression was in accordance with the enzyme changes among the four groups. So the mechanism underlying the decrease in the activities of the lipolytic enzyme following SCC consumption was limited

Morifuji M has reported that the dietary OA decreased the level of serum TG [42]. In the present study, the dietary OA suppressed the serum TG and TC levels, but SCC intake significantly raised the depression (Table 4). This result manifested that dietary SCC may contribute to regulating the assembly and secretion of lipoprotein in liver. MTP, a microsomal lumenal protein, is primarily responsible for transfer of TG and other lipids from their site of synthesis in the endoplasmic reticulum into the lumen during the assembly of VLDL [43-45]. VLDL produced by the liver are the major source of LDL in plasma. So the increase of MTP activity and expression accelerates the secretion of hepatic lipids in the form of lipoprotein. Evidence in this study displayed that OA supplementation significantly inhibited the MTP activity, which also explained the lipid accumulation in liver induced by dietary OA; While the SCC addition to the fatty liver rats elevated the MTP activity (Figure 5), which positively related to the increased serum lipid levels and the decreased hepatic TG concent in the SCC-feeding rats.Thus, this finding led to the suggestion that the enhancement of TG secretion from liver was another pathway of lipid-lowering mechanism of dietary SCC.

In Gao’s study, the long chain base (LCB) of SCC exert as much effect as the cerebroside itself in lowering hepatic lipid levels in mice, which suggests that the LCB might take responsibility for the regulation of hepatic lipid levels [46]. This result provides elementary references for the relationship based on the structure and the activity of the cerebroside but it still deserves further investigation.

## Conclusions

Supplementation with dietary SCC alleviated OA-induced fatty liver by attenuating TG synthesis through the down-regulation of fatty acid synthesis and improving TG secretion from liver. Findings of the present study also supported that SCC might be useful in the preservation of liver functions. But the relationship between the structure and the activity of the cerebroside still requires further investigation.

## Competing interests

The authors declare that they have no competing interests.

## Authors' contributions

BZ , CX and YW contributed in design, experimental work, analysis and publication of results. BZ and YW were responsible for drafting the manuscript. CX , XH and ZL participated in the design of the study, animal studies and performed statistical analysis. JX and JFW were in charge of the sample preparation and discussing the results. YX participated in the design of the study and statistical analysis. CX and TY participated in drafting the manuscript, discussion of results and providing funding for the study. All authors have read and approved this manuscript.
